# Foodservice operator and implementation support practitioner needs for sustaining healthy food retail practices in Australian foodservice settings: findings from a national cross-sectional survey

**DOI:** 10.1186/s12966-026-01944-7

**Published:** 2026-08-05

**Authors:** Bettina Backman, Shaan S Naughton, Vicki Brown, Serene Yoong, Miranda R Blake

**Affiliations:** 1 Faculty of Health, Deakin Institute for Health Transformation, Deakin Centre for Global Preventive Health and Nutrition, School of Health and Social Development, Deakin University, Geelong, Australia; 2 Faculty of Health, Deakin Institute for Health Transformation, Deakin Health Economics (DHE), School of Health and Social Development, Deakin University, Geelong, Australia

**Keywords:** Consumer nutrition environment, Food industry, Health promotion, Nutrition policy, Implementation science

## Abstract

**Background:**

Food outlet practices that support consumers in making healthier food and drink purchases (e.g. healthy product availability and promotion) are key strategies for improving population diets. To have lasting population health impacts, these practices must be sustained. This study explored the perspectives of foodservice operators (e.g. food outlet owners, managers and staff) and practitioners who support them (e.g. health promotion officers and dietitians) on: (i) which healthy food retail practices are typically sustained or discontinued in foodservice contexts; (ii) key determinants influencing sustainment; (iii) support needs for sustaining change; and (iv) differences between sustainers and discontinuers.

**Methods:**

A national cross-sectional online survey was conducted with foodservice operators and implementation support practitioners. The survey was co-designed with representatives from Australian government health departments and health promotion agencies responsible for healthy food retail policies and/or implementation support. Recruitment targeted food retail in public settings (e.g. healthcare food retail, school canteens) and private outlets via Qualtrics panels, professional networks and social media. Survey questions were informed by the Integrated Sustainability Framework, the sustainment-explicit Expert Recommendations for Implementing Change glossary, and existing evidence in the foodservice context. The survey assessed sustained and discontinued practices, determinants of sustainment, and sustainment support needs. Participants were asked to rank selected determinants and support options to indicate relative importance. Descriptive analyses and exploratory chi-square tests were used.

**Results:**

Of 327 eligible participants (234 foodservice operators, 93 practitioners), 212 reported having experience (sustainment or discontinuation) with healthy food retail practices. Among these, 74% reported at least one sustained and 62% at least one discontinued practice. Increasing the range of healthier products was most sustained (*n* = 85), while reducing less healthy products was most often discontinued (*n* = 59). Top-ranked determinants of sustainment were related to supplier availability, organisational leadership, and profitability. Top-ranked sustainment support needs related to financial viability, staff champions and tailored assistance. The reported determinants and support needs were largely similar across sustainers and discontinuers.

**Conclusions:**

This study provides insight into foodservice operator and practitioner reported determinants of sustainment to support designing sustainment supports at scale, including strategies focusing on the financial viability of healthy food retail and supplier engagement.

**Supplementary Information:**

The online version contains supplementary material available at https://doi.org/10.1186/s12966-026-01944-7.

## Background

Long-term transitions towards health-promoting food environments are needed to reduce diet-related health risk factors and improve population health [1, 2]. Foodservice outlets have an increasingly important influence on population diets given the increased consumption of out-of-home foods and drinks worldwide [3–5]. Foodservice outlets are retail outlets where foods and drinks are purchased for immediate consumption (e.g., cafés and restaurants) [6]. Beyond private settings (e.g., cafés in local shopping strips), foodservice outlets are also found in public, government-owned settings (e.g., hospital cafés, school canteens and sport and recreation centre kiosks) across many high-income countries [7–11]. Although public settings in these countries often have nutrition policies in place guiding the healthiness (i.e., nutritional quality) of foods and drinks available for purchase (“healthy food retail policies”) [12, 13], actual product offerings often remain misaligned with public health goals. Evidence from several high-income countries indicates that foods and drinks high in sodium, sugar and/or saturated fat are predominantly available and purchased in public foodservice settings (e.g., sporting centres [8] and healthcare facilities [9–11]).

Consumer purchasing behaviour in foodservice settings is influenced by food outlet practices, particularly the availability and in-store marketing of foods and drinks (product, placement, price and promotion – the “4Ps of marketing”) [6, 14]. Strategies that apply the “4Ps” to encourage healthier food and drink purchases are considered “healthy food retail practices”. In foodservice, examples include offering a higher proportion of healthier menu items or offering healthier options at a lower price compared to less healthy alternatives. One or more of these practices are often used in healthy food retail interventions, such as government healthy food retail policies [15], due to strong evidence showing they can improve the healthiness of consumer food and drink purchases [16–18].

In Australia, healthy food retail policies are commonly in place to support increased availability of and access to nutritious foods and drinks in public settings, with some policies being mandatory in specific contexts (e.g., food available for purchase in school canteens or healthcare settings in some states) [19]. Therefore, foodservice operators in Australian public settings may be required or encouraged to implement healthy food retail practices. These setting- and state-specific healthy food retail policies also include detailed guidance on what constitutes a healthier food or drink, often categorising products as ‘Green’ (healthiest; e.g., fresh fruit), ‘Amber’ (moderately healthy; e.g., dried fruit) or ‘Red’ (least healthy; e.g., confectionery) [15].

The implementation of healthy food retail practices must be *sustained* over time to have long-term benefits on population health [20]. Sustainment refers to the continuation of intervention components after implementation [21, 22]. However, global evidence on whether and how healthy food retail practices in foodservice settings are sustained over long periods of time (greater than 2 years) is limited [16, 23]. A small number of studies have examined short to medium-term sustainment (less than 2 years). For example, Dresser et al. assessed whether healthy food retail practices in New York grocery settings were maintained one year after intervention and found that practices were at risk of being discontinued without additional support and reinforcement [24]. Failed implementation or sustainment may also reduce retailer willingness to re-engage with healthy food retail practices in the future [25].

Successful sustainment of healthy food retail practices requires identifying and addressing key *determinants of sustainment* (i.e., barriers and enablers). *Sustainment strategies* (e.g., ongoing monitoring, tailored support) are needed to address identified determinants (e.g., foodservice operator capacity, and lack of monitoring) [26]. Though sustainment determinants are increasingly studied in grocery and convenience store retail [24, 27–31], evidence from foodservice settings is sparse and mostly limited to follow-ups of single interventions [32–34]. Although context-specific insights are important, identifying common determinants across settings is needed for designing more efficient, centralised sustainment support systems.

Considering the perspectives of foodservice operators and implementation support practitioners provides a comprehensive understanding of what is required for long-term sustainment [35, 36]. In Australian public settings, foodservice business owners, managers and outlet staff (“foodservice operators”) are responsible for daily operations and for sustaining healthy food retail practices. Implementation support practitioners (“practitioners” e.g., local government health promotion officers, healthcare dietitians) typically provide guidance, training and monitoring [37]. Each group is likely to face distinct barriers and enablers influencing the sustainment of healthy food retail practices. Foodservice operators may need ongoing practical support [38], while practitioners may be constrained by time and funding [37].

Several important gaps remain in understanding sustainment within foodservice settings. Existing evidence from original studies is largely limited to narrow contexts, with no large-scale assessment of what drives long-term sustainment across different foodservice contexts. Prior research has predominantly focused on identifying determinants of sustainment, yet little is known about the specific sustainment support needs of foodservice operators. Current evidence also offers limited insight into which types of healthy food retail practices (e.g., product availability vs. pricing) are more or less likely to be sustained. Furthermore, no studies have compared perceived barriers and enablers between foodservice operators who sustained practices and those who discontinued them. These evidence gaps can hinder the development of efficient, system-level strategies to support long-term healthy food retail practice in foodservice outlets.

This exploratory study aimed to describe the perspectives and experiences of foodservice operators and practitioners on sustaining healthy food retail practices in Australian foodservice settings. Specifically, the study explored foodservice operator and practitioner perspectives on (i) which healthy food retail practices are most often sustained or discontinued after initial implementation, (ii) the key determinants (barriers and facilitators) influencing sustainment, (iii) foodservice operator perspectives on their sustainment support needs, and (iv) differences in selected determinants and support needs between sustainers and discontinuers.

## Methods

A cross-sectional online survey was conducted with foodservice operators and practitioners across all Australian states and territories. This study formed a component of a larger national assessment of healthy food retail implementation and sustainment experiences and support needs. This manuscript reports findings from survey components designed a priori to address sustainment, while other components of the national study are reported separately (*manuscript in preparation*). The study was reported in line with the Consensus-Based Checklist for Reporting of Survey Studies [39].

### Sampling and eligibility

A purposive convenience sampling strategy was used. Eligible participants were foodservice operators (e.g., owners, managers, staff or volunteers) and practitioners (e.g., health promotion officers, dietitians, nutritionists) working in or supporting foodservice outlets, aged 18 years or over, and able to understand and write English. Large quick-service chains (more than 20 outlets) were ineligible, as they generally operate through centralised business models (limited outlet-level decision-making) and are characterised by predominantly unhealthy business practices in the Australian context [40]. Food manufacturers, settings where customers do not purchase food (e.g., charity kitchens), and practitioners with no experience in supporting foodservice outlets were also ineligible.

No formal sample size calculation was undertaken for this study as analyses were primarily exploratory and descriptive. Recruitment aimed to achieve broad representation of foodservice operators and practitioners across priority public settings for promoting population health (e.g., public hospitals, sports and recreation settings, school canteens) [41, 42], and across all Australian states and territories.

### Recruitment and data collection

Participants were recruited through two main channels from May to July 2025: (i) purposefully distributing recruitment advertisements via state government representatives, health promotion services (e.g., the Healthy Eating Advisory Service [43]), and other professional networks, and social media (LinkedIn), and (ii) targeted panel recruitment through Qualtrics. Qualtrics panel recruitment involves sourcing participants from pre-recruited panels who have agreed to participate in survey research and can be screened based on study-specific criteria [44]. Only foodservice operators were recruited through Qualtrics due to difficulties in reaching this group via standard recruitment methods [45]. Recruitment focused primarily on public settings; however, private foodservice operators were also invited via the Qualtrics-distributed survey. The cross-sectional survey was administered via Qualtrics and took approximately 15 min to complete. To prevent duplicate submissions, Qualtrics’ fraud detection items were used, including checks for duplicate IP addresses, and survey entries submitted simultaneously with identical response patterns. Participants recruited via professional networks and social media were offered the opportunity to enter a random number draw to win one of five AUD$50 gift vouchers. Qualtrics panel participants received reimbursement directly through Qualtrics.

#### Surveys and measures

The survey (Supplementary Material 1) was co-designed with representatives from Australian Departments of Health and state preventive health agencies to enhance engagement, ensure policy and practice relevance, and support the face validity of survey items. Representatives reviewed draft questions, suggested additional content, and supported recruitment through jurisdictional communication channels. The survey was iteratively pre-tested and refined by the research team, and pilot-tested with three members of the broader research team (two researchers and one communications specialist) prior to data collection.

Survey items were informed by existing literature and relevant theoretical frameworks, and their wording and final selection were refined through the co-design process. The items included closed- and open-ended questions to assess: (i) participant eligibility (e.g., “Do you own, manage, work or volunteer in a food outlet that mainly sells food for immediate consumption (e.g. a café, kiosk, or canteen)?”; see Supplementary Material 1), (ii) demographic and contextual information (e.g., participant role, outlet characteristics, foodservice operator healthy food retail experience); (iii) commonly sustained and discontinued practices (according to the “4Ps” [46], see Supplementary Material 1); (iv) barriers and enablers to sustainment, informed by the Integrated Sustainability Framework [47] and existing literature [19, 25, 48] (including our recent systematic review of determinants of sustained healthy food retail interventions [49]); and (iv) support needs for sustainment, informed by the Expert Recommendations for Implementing Change (ERIC) glossary [26].

Two versions of the survey were distributed: a combined foodservice operator-practitioner survey and a separate foodservice operator-only survey administered via Qualtrics. Both versions used branching logic to tailor question pathways based on participant role and foodservice operator experience with healthy food retail practices. For foodservice operators without prior experience implementing healthy food retail practices, question wording was adapted to reflect perceived relevance, and foodservice operators reporting neither experience nor interest were not asked questions related to support needs.

##### Demographic and contextual information

Demographic and contextual information were collected using categorical (e.g. jurisdiction and participant role) and open-text response options (e.g. postcode or local government area). Participant postcode was used to assess area-level socioeconomic disadvantage using the Australian Socio-Economic Indexes for Areas Index of Relative Socioeconomic Disadvantage (SEIFA IRSD) [50]. Postcode-level IRSD deciles were categorised into higher socioeconomic disadvantage (deciles 1–5) and lower socioeconomic disadvantage (deciles 6–10). Foodservice operators’ experience with healthy food retail practices was assessed using the question: “Do you have experience making or attempting to make changes in your food outlet to encourage customers to choose healthier foods or drinks?”. Categorical response options included: “Yes, I have experience”, “No, but we are interested”, and “No, and we are not currently interested”.

##### Healthy food retail practices

Healthy food retail practices were measured across the “4Ps” relevant to foodservice context: product (e.g. increasing healthier options, reducing less healthy items, and improving recipes), placement (e.g. making healthier items more prominent), pricing (e.g. discounts on healthier items or increasing prices for less healthy options), and promotion (e.g. introducing or removing promotional materials to support healthier choices) (see Supplementary Material 1). Sustained and discontinued practices were measured using a response matrix, with each practice type rated using four response options (not attempted, discontinued, implemented recently, sustained). Given that practitioners often work across multiple outlets with varying sustainment experience, they were asked to reflect on their overall experience across outlets.

##### Determinants of sustained healthy food retail practices

Barriers and enablers were assessed using a similar matrix format (see Supplementary Material 1), with each prespecified determinant rated using three response options (enabler/barrier to implementation; enabler/barrier to sustainment; not sure/not relevant). Barriers and enablers were assessed separately. Participants then ranked each selected sustainment-related enabler and barrier in order of importance (1 = most important; up to 10 items). Foodservice operator support needs were assessed using categorical options and optional free-text fields. Foodservice operators with experience or interest in implementing healthy food retail practices then ranked selected support needs in order of helpfulness to sustainment (1 = most helpful; up to 5 items). Free-text responses were re-coded by the lead author where possible to align with pre-defined categorical options; responses that could not be re-coded were classified as “other”. “Sustaining” in the context of this study was defined as the continued delivery of healthier food retail practices over time following their initial implementation. The sustainment timeframe was not defined in the survey to minimise respondent burden and maintain survey simplicity, given that sustainment was one of several constructs assessed.

### Statistical analysis

The data were analysed using Microsoft Excel and Stata (version 18). Participants with demographic-only responses (i.e., responses up to the first 10 questions in the survey; see Supplementary Material 1), or representing ineligible roles (e.g., food manufacturer) or settings (e.g., charity kitchens) were excluded from the analysis. Only responses relevant to sustainment were included in the analysis of determinants; participants who reported implementation determinants only, without sustainment-related responses, were excluded. Participants with missing data were excluded from specific analyses where required variables were unavailable, resulting in varying sample sizes across analyses. As this was an exploratory study, responses were not required to be complete for inclusion. Participants were included in analyses where sufficient data were available, with at least one response to items relevant to the specific analysis required for inclusion. Sample sizes for each analysis are reported in the Results.

#### Descriptive analyses

Descriptive statistics were used to summarise participant characteristics, outlet and area-based details, and the prevalence of sustained or discontinued healthy food retail practices across practice types (product availability, recipe modification, placement, pricing and promotional materials). Descriptive analyses were also conducted for all determinants (barriers and enablers) of sustainment and sustainment support needs reported by participants. Proportions were used to estimate the prevalence of each determinant, and 95% confidence intervals were reported to indicate precision and assist interpretation. Descriptive analyses of ranking responses were used to identify priority determinants and foodservice operator sustainment support needs. Proportions were calculated for how often each item was ranked in the top three to identify most important determinants and supports.

#### Group comparisons

Chi-square tests were used to explore potential differences in the mean proportions of selected determinants and supports between sustainers and discontinuers. Sustainers were defined as foodservice operators who sustained product availability practices (“increased availability of healthy food/drinks” and/or “reduced availability of less healthy food/drinks”), or sustained one practice and never attempted the other. Discontinuers were foodservice operators who discontinued either practice, regardless of whether the other was sustained. This categorisation was based on the key focus on product availability in Australian state government healthy food retail policies, which typically focus on increasing the relative availability of healthier foods and drinks [15]. Interpretation focused on differences in mean proportions, with 95% confidence intervals and p-values (*p* < 0.05). Given the exploratory nature of the study, group comparisons were conducted without pre-specified hypotheses.

Additional exploratory analyses were conducted to explore differences in proportions selecting enablers, barriers and supports between foodservice operators with experience implementing healthy food retail practices and those without experience (but who were interested in implementing).

#### Sensitivity analyses

Survey analyses were repeated after excluding responses flagged as ‘lower quality’ (identified through response inconsistencies and short completion times, confirmed by the research team) to assess the robustness of survey findings.

## Results

### Participant characteristics

**Fig. 1:**
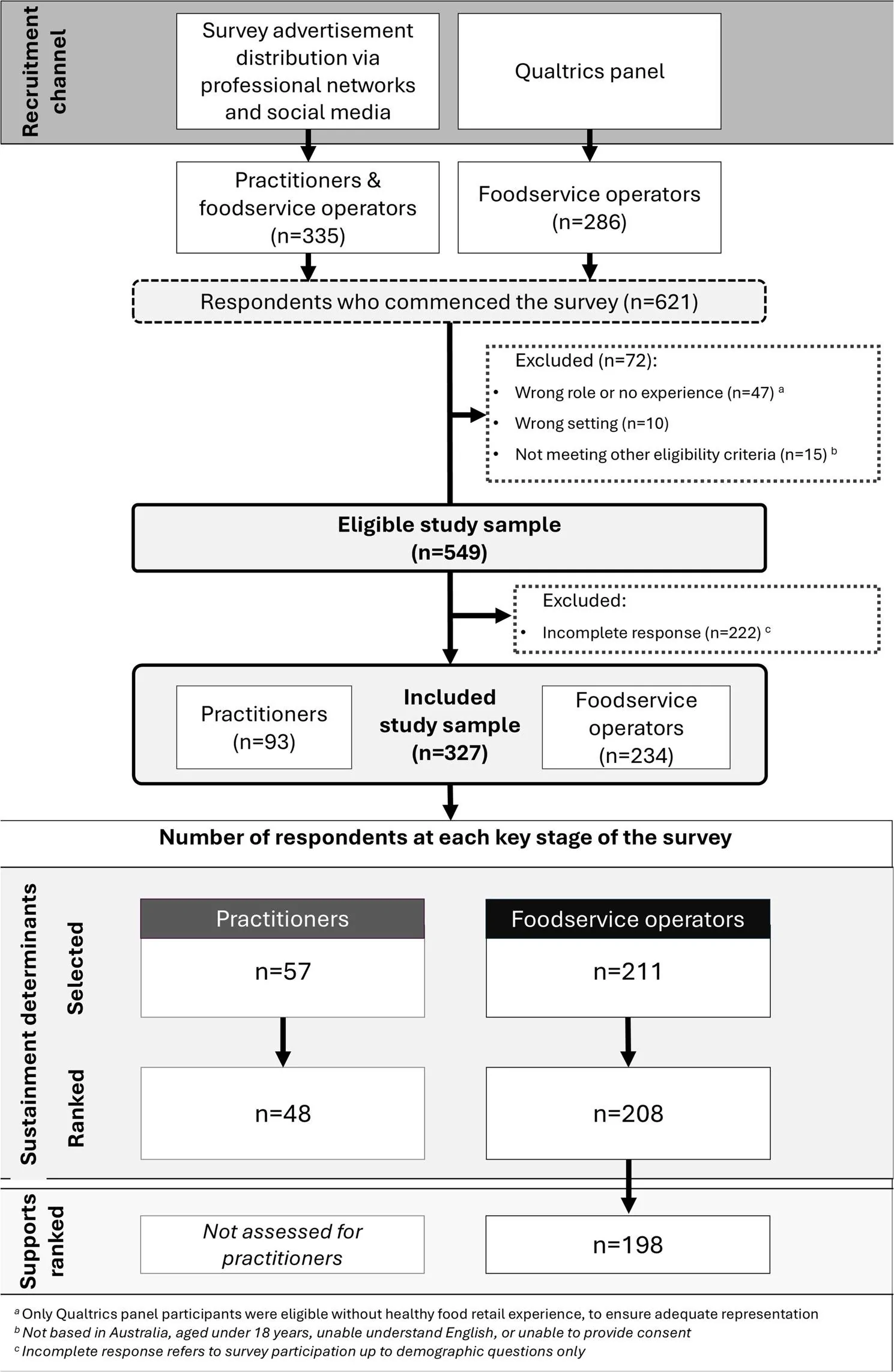
Participant recruitment and inclusion in the healthy food retail sustainment study (flow diagram). The diagram shows recruitment channels, eligibility screening, and the number of practitioners and foodservice operators included at each stage of the survey and analysis

Of the 608 individuals who began the survey, 327 were eligible for this study (234 foodservice operators and 93 practitioners; Fig. 1). Most exclusions were due to responses up to demographic questions only (*n* = 222), followed by ineligible roles (*n* = 47; e.g., food manufacturers), ineligible settings (*n* = 10; e.g., charity kitchens), and other reasons (e.g., not based in Australia; *n* = 2). Most foodservice operators (*n* = 216) were recruited through Qualtrics, while all practitioners were recruited via professional and jurisdictional networks. Among eligible participants, the proportion of completed survey questions ranged from 11% to 100% (mean 89%, SD 27), with 83% (*n* = 272) completing all questions displayed to them. Figure 1 provides detailed numbers of respondents at each stage of the analysis.

Table 1 summarises participant characteristics. Participants represented all Australian states and territories, with the largest proportions from the most populous states of Victoria (31%) and New South Wales (22%). Both higher (53%) and lower socioeconomic disadvantage (45%) areas were represented (IRSD data was not available for 2% of participants). The most frequently reported settings were schools and sports clubs (each 31%), and 44% of participants (*n* = 144) reported working across multiple settings. Among those working in a single setting (mostly foodservice operators), private food outlets were most common (*n* = 44). Most participants (80%, *n* = 261) had experience implementing or supporting healthy food retail practices (all practitioners and 72% of foodservice operators). Of the remaining foodservice operators (*n* = 66), most expressed interest in healthy food retail practices (86%; *n* = 57), while nine (14%) were not currently interested.

**Table 1:** Healthy food retail sustainment survey participant characteristics

			Foodservice operators			Practitioners			Total study sample *n* (%)
			*n*	% of retailers	% of study sample	*n*	% of practitioners	% of study sample	
Total			234	-	72%	93	-	28%	327
State	Australian Capital Territory		5	2%	2%	1	1%	0%	6 (2%)
	New South Wales		59	25%	18%	12	13%	4%	71 (22%)
	Northern Territory		0	0%	0%	7	8%	2%	7 (2%)
	Queensland		47	20%	14%	19	20%	6%	66 (20%)
	South Australia		23	10%	7%	5	5%	2%	28 (9%)
	Tasmania		7	3%	2%	7	8%	2%	14 (4%)
	Victoria		69	29%	21%	31	33%	10%	100 (31%)
	Western Australia		24	10%	7%	11	12%	3%	35 (11%)
SEIFA (IRSD)^a^	Higher socioeconomic disadvantage (deciles 1–5)		126	54%	39%	48	51%	15%	174 (53%)
	Lower socioeconomic disadvantage (deciles 6–10)		107	46%	33%	40	43%	12%	147 (45%)
	Data not available		1	0.5%	0.3%	5	5%	2%	6 (2%)
Food retail setting^b^	Schools		41	18%	13%	60	65%	18%	101 (31%)
	Sports clubs		53	23%	16%	47	51%	14%	100 (31%)
	Hospital and health service facility^c^		49	21%	15%	50	54%	15%	99 (30%)
	Workplace		56	24%	17%	38	41%	12%	94 (29%)
	Sports and recreation setting (e.g., aquatic centre, stadium)		42	18%	13%	46	49%	14%	88 (27%)
	Private settings (e.g. restaurant)		50	21%	15%	11	12%	3%	61 (19%)
	Arts, cultural or events setting		29	12%	9%	30	32%	9%	59 (18%)
	Park		26	11%	8%	12	13%	4%	38 (12%)
	University		19	8%	6%	6	6%	2%	25 (8%)
	Other (e.g., transport settings)		4	2%	1%	8	9%	2%	12 (4%)
Role	Foodservice operator	Staff (customer facing and/or food preparation)	72	31%	-	-	-	-	22%
		Food outlet manager	56	24%	-	-	-	-	17%
		Food outlet volunteer	50	21%	-	-	-	-	15%
		Food outlet owner	32	14%	-	-	-	-	10%
		Food outlet shift supervisor	18	8%	-	-	-	-	6%
		Other food outlet role (e.g. delivery driver)	5	2%	-	-	-	-	2%
	Practitioner	Environmental health officer	-	-	-	35	38%	-	11%
		Health promotion officer	-	-	-	34	37%	-	10%
		Nutritionist or dietitian	-	-	-	17	18%	-	5%
		Other practitioner role (e.g., local government officer)	-	-	-	7	8%	-	2%

This table presents participant characteristics from an Australian national healthy food retail sustainment survey conducted between May and July 2025. Participants included foodservice operators and implementation support practitioners. Percentages are reported within subgroups and for the total study sample

^a^ SEIFA (IRSD) values reflect the Index of Relative Socioeconomic Disadvantage for the participant’s work location. For participants working across multiple local government areas, the lowest available decile was used. IRSD data were unavailable for six participants (1 foodservice operator, 5 practitioners)

^b^ Participants could select more than one type of food retail setting; therefore, percentages may sum to more than 100%

^c^ While charitable organisations (e.g., foodbanks) were generally excluded from this study, hospital auxiliary outlets were included as they represent typical food outlets within healthcare settings in some states (e.g., Queensland)

### Sustainment of healthy food retail practices

**Fig. 2:**
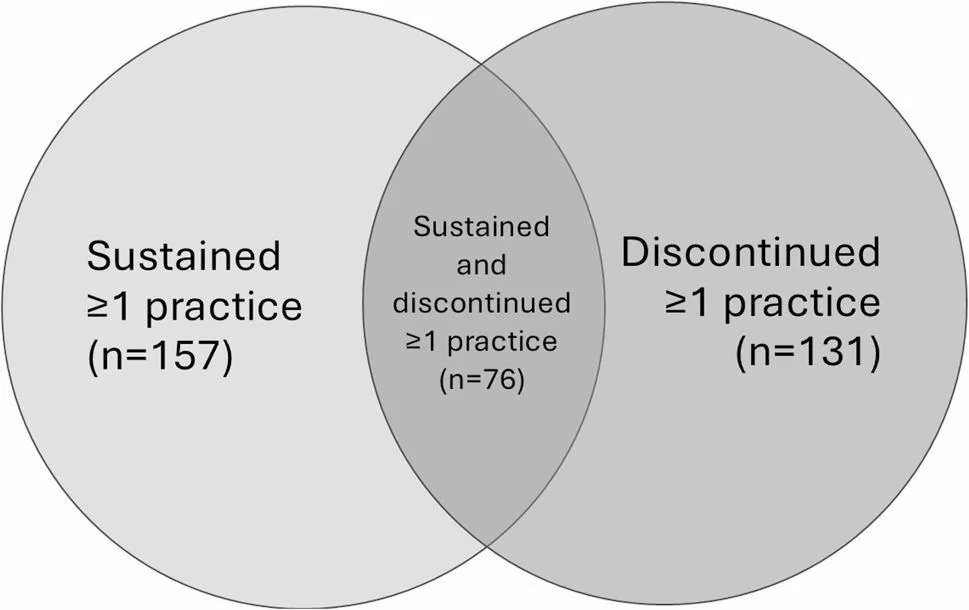
Overlap between participants who sustained and/or discontinued at least one healthy food retail practice . The diagram shows the overlap between participants who reported sustaining at least one practice, discontinuing at least one practice, or both. The total sample includes participants who reported either sustained or discontinued practices (*n*=212)

Overall, 212 participants (65% of the sample) reported either sustainment or discontinuation of at least one healthy food retail practice. Of these, 157 participants (74% of the sample [95% CI: 68%, 80%]; 48% of the total sample) reported sustaining at least one practice, while 131 (62% [55%, 68%]; 40% of the total sample) reported discontinuing at least one practice (Table 2). Confidence intervals indicated no meaningful differences in the proportions of foodservice operators versus practitioners reporting sustainment or discontinuation. Overall, 76 participants reported both sustaining and discontinuing at least one healthy food retail practice (Fig. 2).

**Table 2:** Sustained and discontinued healthy food retail practices reported by survey participants

Healthy food retail practice	Total *n* ^a^	Sustained							Discontinued						
		All			Foodservice operators		Practitioners		All			Foodservice operators		Practitioners	
		n	%	95% CI	%	95% CI	%	*95% CI*	*n*	%	95% CI	%	95% CI	%	95% CI
At least one practice sustained or discontinued	212	**157**	**74**	**68**,** 80**	74	67, 81	73	61, 83	**131**	**62**	**55**,** 68**	60	51, 67	67	55, 78
Increased range of healthy food	122	**85**	**70**	**62**,** 78**	70	59, 80	70	56, 83	**37**	**30**	**22**,** 38**	30	20, 41	30	17, 44
Reduced range of less healthy food	118	**59**	**50**	**41**,** 59**	45	34, 57	58	43, 72	**59**	**50**	**41**,** 59**	55	43, 66	42	28, 57
Recipe modifications (to improve healthiness)	100	**65**	**65**	**56**,** 74**	65	54, 76	65	48, 81	**35**	**35**	**26**,** 44**	35	24, 46	35	19, 52
Product placement	109	**75**	**69**	**60**,** 78**	70	60, 80	66	49, 82	**34**	**31**	**22**,** 40**	30	20, 40	34	18, 51
Pricing strategies	82	**39**	**48**	**37**,** 58**	52	39, 64	33	12, 55	**43**	**52**	**40**,** 62**	48	36, 61	67	45, 88
New promotional materials or activity on healthy products	93	**59**	**63**	**54**,** 73**	58	47, 71	75	59, 91	**34**	**37**	**27**,** 46**	42	30, 54	25	9, 41
Removal of less healthy product promotional materials or activity	104	**65**	**63**	**53**,** 72**	63	51, 74	63	46, 79	**39**	**38**	**28**,** 47**	38	26, 49	38	21, 54

This table presents the proportion of foodservice operators and implementation support practitioners reporting sustained or discontinued healthy food retail practices in Australian foodservice settings. Results are shown overall and stratified by participant group. Percentages represent the proportion of survey participants reporting sustainment or discontinuation of each practice, with 95% confidence intervals (CI). Participants could report more than one practice

^a^ Total *n*= participants reporting either sustainment and/or discontinuation of any healthy food retail practice. Bolded values indicate totals across both participant groups (foodservice operators and practitioners)

**Fig. 3:**
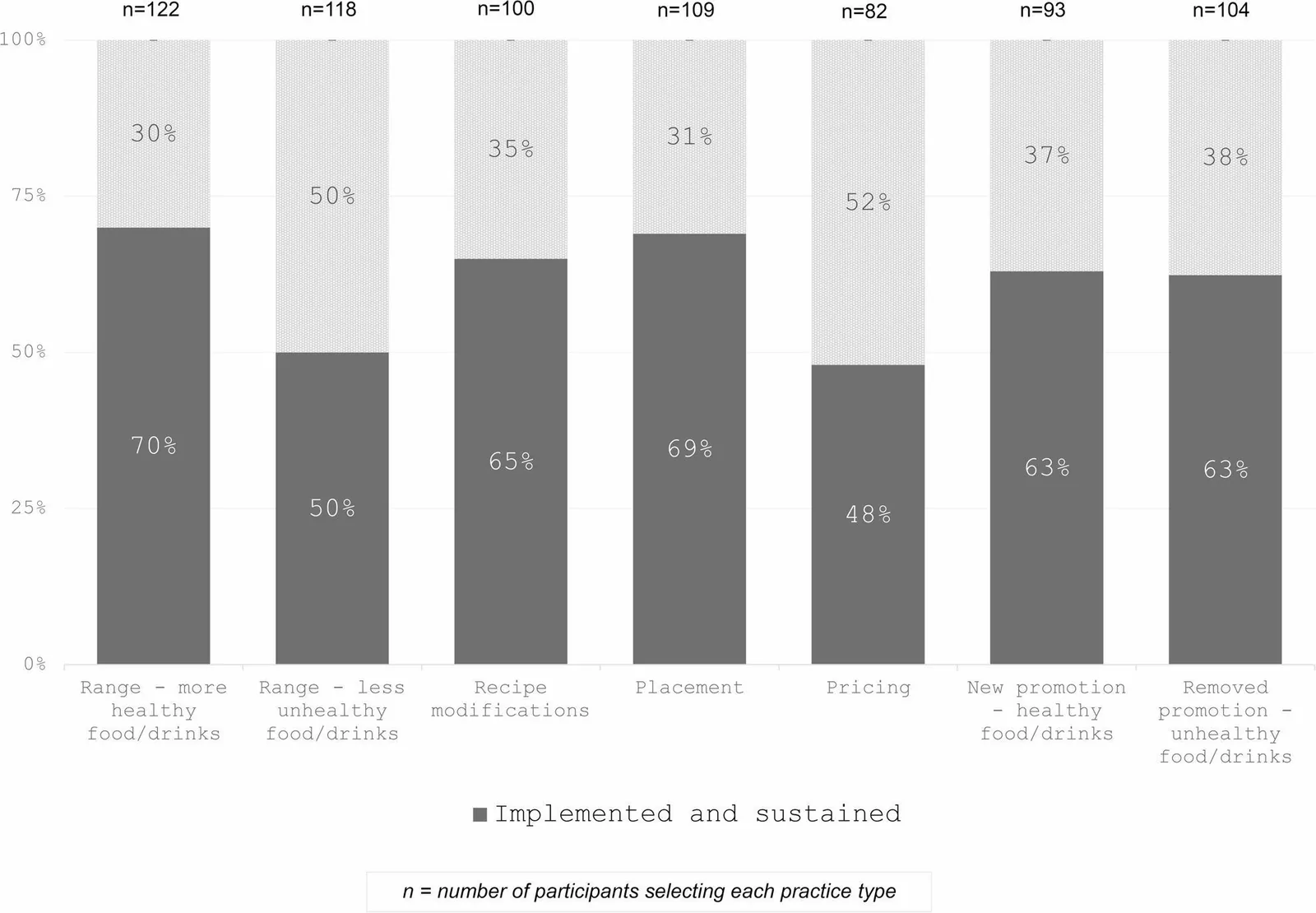
Self-reported sustainment of healthy food retail practices in Australian foodservice settings. Proportion of survey participants (foodservice operators and practitioners) reporting sustained (“implemented and sustained”) or discontinued (“implemented but discontinued”) healthy food retail practices in food outlets they operate or support. Bars show the percentage of participants reporting each outcome by practice type that they had attempted

On average, participants reported three sustained practices and two discontinued practices. A small number of participants reported sustaining (*n* = 18) or discontinuing (*n* = 14) all practices after initial implementation. The most frequently sustained practice was increasing the range of healthier food and/or drinks, reported by 70% (95% CI: 62%, 78%; *n* = 85) of participants with experience in this practice (Fig. 3). The least sustained practices were pricing strategies (*n* = 39; 48% [95% CI: 37%, 58%]) and reducing the range of less healthy foods and/or drinks (*n* = 59; 50% [41%, 59%]). Reducing the range of less healthy items was also the most frequently discontinued practice. Pricing strategies had the highest discontinuation rate (52% [40%, 62%]) among participants with experience in this practice (*n* = 82). No notable differences were observed between practitioners and foodservice operators in which practices were more likely to be reported as sustained or discontinued (Table 2). Additionally, 11 participants reported both sustaining and discontinuing product availability-related practices (e.g., sustaining increased availability of healthier options while discontinuing reduced availability of less healthy items).

### Enablers and barriers to sustained changes

#### Foodservice operators

Among foodservice operators who reported enablers and barriers to sustained changes (*n* = 211; 90% of the foodservice operator sample) 202 (86%) ranked their selected barriers and 208 (89%) ranked enablers. Foodservice operators selected a median of six enablers (of 11 listed) and seven barriers (of 15 listed, including open-text “other” options), and ranked a median of five items in each category.

The two most frequently reported enablers were “*Monitoring and evaluation of healthy changes made*” (72% [95% CI: 66%, 77%]) and “*Healthy food and supplier availability*” (69% [63%, 75%]; Table 3). When asked to rank the enablers by perceived importance, *supplier availability* was most frequently ranked in the top three (36% [30%, 43%]), followed by “*My food outlet has a clear obligation to provide healthy food and drink options*” (34% [28%, 40%]) and “*Food outlet manager’s and/or staff’s willingness or motivation to make healthy changes”* (30% [24%, 36%]).

**Table 3:** Determinants of sustained healthy food retail practices selected and ranked by foodservice operators

Determinant	% selecting determinant (95% CI)^a^	% ranking determinant in top 3 (95% CI)^b^	Selection difference (mean % difference, 95% CI)
Enablers	*n* = 211	*n* = 208	Discontinuers(*n* = 48) v. sustainers (*n* = 57)
Monitoring and evaluation of healthy changes made	72 (66, 78)	26 (20, 32)	-2 (-20, 14)
Healthy food and supplier availability	69 (63, 75)	36 (30, 43)	-16 (-33, 1)
My food outlet has a clear obligation to provide healthy food and drink options	66 (60, 72)	34 (28, 40)	-8 (-26, 11)
Food outlet manager’s and/or staff’s willingness or motivation to make healthy changes	66 (59, 72)	30 (24, 36)	-10 (-28, 9)
Customers purchase or request healthy options	63 (56, 69)	28 (22, 34)	-8 (-27, 11)
My food outlet manager’s and/or staff’s skills and knowledge	62 (56, 68)	24 (18, 30)	+ 9 (-10, 28)
My and/or my food outlet’s previous experience making healthy changes	57 (51, 64)	21 (16, 27)	-7 (-27, 12)
Staged implementation strategies (e.g., adding healthier items incrementally or before removing less healthy items)	55 (49, 62)	22 (17, 28)	+ 10 (-9, 29)
Availability of useful resources (e.g., checklists, guidelines, planograms, pre-tested recipe ideas)	55 (48, 61)	21 (16, 27)	**-22 (-40**, -4) *
Dedicated technical support (e.g., someone who can provide advice on why an item is classified as it is, and suggest potential alternatives)	50 (43, 57)	20 (15 ,26)	+ 10 (-9, 30)
Barriers	*n* = 211	*n* = 202	Discontinuers (*n* = 49) v. sustainers (*n* = 54)
Additional costs and/or profit losses associated with healthy changes	59 (52, 65)	26 (21, 33)	-16 (-35, 4)
My food outlet, manager and/or staff are resistant to making healthy changes	59 (52, 65)	15 (11, 20)	-13 (-32, 6)
Burden and/or additional workload associated with healthy changes	57 (51, 64)	19 (14, 25)	**+ 25 (5**, 44) *
Difficulty accessing healthier options or ingredients from suppliers	55 (49, 62)	25 (19, 31)	+ 17 (-2, 36)
Lack of access to dedicated support (from individuals or organisations)	55 (49, 62)	24 (18, 30)	+ 1 (-19, 21)
Limited access to practical resources (e.g., implementation support tools, checklists, guidelines, pre-tested recipe ideas)	55 (48, 61)	16 (11, 22)	+ 1 (-18, 21)
Lack of compliance monitoring or incentives	54 (47, 61)	18 (13, 24)	+ 8 (-28, 11)
Not sure what to do next	53 (46, 60)	11 (7, 16)	+ 3 (-17, 23)
Customer demand for less healthy options (e.g., confectionery)	49 (42, 56)	23 (18, 30)	+ 8 (-11, 28)
Competition between food outlets (e.g., other outlets keep stocking less healthy items or healthier items at lower prices)	48 (42, 55)	22 (17, 29)	+ 14 (-6, 33)
There’s no clear expectation for my outlet to provide healthier food and drinks	47 (41, 54)	13 (9, 19)	**+ 20 (1**, 40) *
Unclear or open to interpretation what counts as a healthy change	45 (38, 52)	12 (8, 18)	**+ 29 (11**, 48) *
Healthy changes have complex or strict rules that must be followed, with little room to adapt them to my outlet	42 (36, 49)	16 (11, 22)	**+ 29 (11**, 47) *
High food outlet staff turnover	37 (31, 44)	20 (15, 26)	**+ 20 (3**, 39) *

This table presents determinants (enablers and barriers) of sustained healthy food retail practices reported and ranked by foodservice operators (*n* = 211) participating in an Australian national survey of healthy food retail support needs. Results include the proportion selecting each determinant, the proportion ranking it among the top three barriers/enablers, and mean proportion differences between comparison groups, along with 95% confidence intervals (CI). Statistically significant findings are shown in bold and marked with an asterisk (*)

^a^ Percentage of foodservice operators selecting at least one enabler (*n*=211) or barrier (*n*=211)

^b^ Percentage of foodservice operators ranking the determinant among their top three based on importance for sustainment (enabler ranking *n*=208; barrier ranking *n*=202)

In terms of barriers, the two most frequently reported barriers were “*Additional costs and/or profit losses associated with healthy changes*” and “*My food outlet*,* manager and/or staff are resistant to making healthy changes*” (both 59% [52%, 65%]). For barriers perceived most important, “*Costs and profit losses”* (26% [21%, 33%]) was the most frequently ranked in top three, followed by “*Difficulty accessing healthier options or ingredients from suppliers*” (25% [19%, 31%]) and “*Lack of access to dedicated support*” (24% [18%, 30%]).

Overall, most frequently reported determinants were largely similar between sustainers and discontinuers (Table 3). However, discontinuers were more likely than sustainers to select “*Burden and/or additional workload*” as a barrier (mean difference [MD] + 25% [5%, 44%]). Exploratory analyses indicated only minor differences in selected and ranked determinants between foodservice operators with and without experience implementing healthy food retail practices (Supplementary Material 2).

### Practitioners

Among practitioners, 57 (59% of the practitioner sample) reported enablers, 54 (56%) reported barriers, and 48 (50%) ranked their selected determinants. Practitioners selected and ranked a median of four (of 11) enablers and five (of 14) barriers.

The most frequently reported and top-three-ranked enabler was “*Strong rapport between retailers* [i.e. foodservice operators] *and those supporting them to make change*” (selected by 74% [60, 84]; ranked by 65%; Table 4). “*Support from the leadership*,* executive or board in the food outlet’s parent organisatio*n” was the second most selected and ranked (53% [40, 68]; 54%). Other enablers were less frequently reported.

**Table 4:** Determinants of sustained healthy food retail practices selected and ranked by implementation support practitioners

Determinant	% selecting determinant (95% CI)^a^	% ranking determinant in top 3 (95% CI)^b^
Enablers	*n* = 57	*n* = 48
Strong rapport between retailers^c^ and those supporting them to make change	74% (60, 84)	65% (50,77)
Support from the leadership, executive or board in the food outlet’s parent organisation	53% (40, 65)	54% (40, 68)
Staged implementation strategies (e.g., adding healthier items incrementally or before removing less healthy items)	44% (31, 57)	29% (18, 44)
Availability of useful resources (e.g., checklists, guidelines, planograms, pre-tested recipe ideas)	44% (31, 57)	21% (11,35)
Support from state or territory funded agencies or peak bodies	40% (28, 54)	27% (16, 42)
Dedicated technical support (e.g., someone who can provide advice on why an item is classified as it is, and suggest potential alternatives)	37% (25, 50)	25% (15,39)
Providing retailers^c^ with resources	35% (24, 49)	10% (4, 23)
My previous experience with supporting retailers to make changes	33% (22, 47)	17% (8,30)
Support and advice from other practitioners	33% (22, 47)	10% (4, 23)
Liaising with suppliers to identify healthier items	25% (15, 38)	10% (4, 23)
Barriers	*n* = 54	*n* = 48
Not enough support from the leadership or executive in the food outlet’s parent organisation	61% (47, 73)	42% (28, 56)
Retailers^c^ concerned about losing customers or money	59% (46, 71)	44% (30, 58)
High staff turnover within the retail setting	57% (44, 70)	35% (23, 50)
Not enough time, capacity or FTE in my current role	48% (35, 62)	33% (21, 48)
Lack of support or communication from the policy setter	44% (32, 58)	25% (15, 40)
Retailers^c^ not having the skills or knowledge to implement changes	43% (30, 56)	19% (10, 33)
Difficulty accessing healthier options or ingredients from suppliers	39% (27, 53)	23% (13, 37)
Retailer^c^ concern about increased workloads	39% (27, 53)	13% (6, 26)
Unclear or open to interpretation what counts as a healthy change	37% (25, 51)	6% (2,18)
Lack of rapport with retailers	33% (22, 47)	10% (4, 23)
Limited access to practical resources (e.g., implementation support tools, checklists, guidelines, pre-tested recipe ideas)	28% (17, 41)	6% (2, 18)
Retailers/staff^c^ feel implementation is prescriptive instead of a collaborative process	28% (17, 41)	2% (0, 14)
Not sure what to do next	26% (16, 40)	6% (2, 18)

This table presents determinants (enablers and barriers) of sustained healthy food retail practices reported and ranked by implementation support practitioners (*n* = 57) participating in an Australian national survey of healthy food retail support needs. Results include the proportion selecting each determinant, the proportion ranking it among the top three barriers/enablers, and mean proportion differences between comparison groups, along with95% confidence intervals (CI)

^a^Percentage of practitioners selecting at least one enabler (*n* = 57) or barrier (*n* = 54)﻿

^b^Percentage of implementers ranking the determinant among their top three based on importance for sustainment (*n* = 48)

^c^ Retailer = foodservice operator

The most frequently reported and ranked barriers were “*Not enough support from the leadership or executive in the food outlet’s parent organisation*” (61% [47%, 73%]; ranked by 42%) and “*Retailers concerned about losing customers or money*” (59% [46%, 71%]; ranked by 44%). “*High staff turnover within the retail setting*” was the third commonly reported barrier (57% [44%, 70%] ranked by 35%).

### Sustainment support needs from the foodservice operator perspective

A total of 198 foodservice operators (85% of the foodservice operator sample) ranked sustainment support options. The most frequently top-three-ranked supports were “*Support with making money by selling healthy options*” (21% [16%, 27%]), “*Help identifying and training a staff member to lead changes”* (17% [12%, 23%]), and “*Support with planning healthy changes in a way that suits the outlet*” (16% [12%, 22%]) (Table 5). No differences in reported sustainment support needs were observed between sustainers and discontinuers. Some small differences were observed between foodservice operators with and without healthy food retail experience (e.g., foodservice operators without experience less frequently selected “*Support identifying new suppliers”;* see Supplementary Material 3).

**Table 5:** Foodservice operator-preferred support options for sustaining healthy food retail practices

Support option	% ranking determinant in Top 3 (95% CI)^a^ *n* = 198	Mean difference in % ranking determinant in Top 3 (95% CI)
		Discontinuers (*n* = 52) v. sustainers (*n* = 48)^b^
Making money by selling healthy options	21 (16, 27)	-2 (-19, 15)
Help identifying and training a staff member to lead healthy changes in my outlet	17 (12, 23)	-9 (-24, 7)
Support to plan healthy changes in a way that works for my outlet	16 (12, 22)	+ 7 (-6, 19)
Access to external funding or financial incentives to support healthy changes	15 (11, 21)	-10 (-25, 4)
Improving the skills of the staff to prepare healthier options	15 (11, 21)	1 (-11, 13)
Recipe modifications to deal with food allergies and intolerance while meeting nutrition standards	15 (10, 20)	-13 (-27, 1)
Engaging customers in healthy options	15 (10, 20)	+ 5 (-9, 20)
Support with getting feedback from customers	14 (10, 19)	-5 (-19, 10)
Advice or support on labelling (e.g., traffic light labelling/health stars/ ‘everyday’ or ‘occasional’ labelling, high or low in a nutrient) or naming of menu items	14 (10, 19)	-1 (-15, 13)
Support identifying new or alternate healthy products	13 (9, 19)	-9 (-23, 5)
Advice on food or financial waste management	12 (8, 17)	-3 (-16, 10)
Resources to promote the healthy food options to customers	12 (8, 17)	+ 1 (-13, 15)
Support identifying new suppliers	11 (7, 16)	+ 1 (-12, 14)
Feedback on how my food outlet is performing against nutrition/food policy targets	11 (7, 16)	-3 (-14, 7)
Support to connect with other retailers who have successfully made healthy changes	11 (7, 16)	-3 (-15, 9)
Advice on setting the everyday price of items	11 (7, 16)	+ 9 (-3, 21)
Advice on price promotions or special deals	10 (7, 15)	-1 (-14, 11)
Help auditing the healthiness of our food environment, product offerings, or compliance with policy	10 (6, 15)	+ 7 (-5, 18)
New equipment to prepare, store or display healthy foods and/or drinks	8 (5, 13)	+ 9 (-2, 19)
Advice on planograms or stock display	6 (3, 10)	-1 (-12, 9)
Advice on marketing or advertising for the food outlet to customers	6 (3, 10)	0 (-8, 8)

This table presents support options that retailers reported wanting to access and perceived as helpful for sustaining healthy food retail practices in Australian foodservice settings. Results show the proportion of retailers ranking each support option among their top three supports for sustainment and mean proportion differences between comparison groups, with 95% confidence intervals (CI)

^a^ Percentage of foodservice operators ranking the support option among their top three based on importance for sustainment (*n*=198)

^b^ Difference in mean proportions between sustainers (*n*=52) and discontinuers of product availability practices (*n*=48). Denominators include only those who ranked at least one support option

### Sensitivity analyses

Sensitivity analyses excluding responses flagged as lower quality (*n* = 29) produced similar results to the primary analyses. The direction and relative importance of sustained and discontinued practices, determinants of sustainment, and sustainment support needs remained unchanged.

## Discussion

This national survey found that increasing the range of healthier products was the most sustained practice, while pricing and product-restriction strategies were less often maintained by participants in Australian food service settings. From a foodservice operators’ perspective, supplier availability and a clear organisational obligation to provide healthier options were key enablers of sustainment. Practitioners highlighted a need for strong rapport with foodservice operators, and leadership support. Both groups reported costs and profit losses as key barriers. Foodservice operators also highlighted the role of supplier availability and practitioners highlighted a lack of organisational support as barriers. Few meaningful differences were observed between sustainers and discontinuers. Foodservice operator-reported sustainment support needs focused on financial viability, staff champions and tailoring practices to fit with the outlet’s context.

Our findings align with previous studies suggesting that supplier availability, financial viability and leadership support are some of the key drivers of sustained healthy food retail interventions [23, 51, 52]. For example, profitability and business impacts have been identified as important determinants across the intervention life cycle, including during sustainment [23]. Recent research in university vending contexts has similarly highlighted the importance of financial viability as a key enabler to healthy vending intervention which was sustained over time [51]. While Blake et al. (2020) [52] found that foodservice operator-perceived profitability concerns in public foodservice settings may lessen over time as customers adapt to healthier offerings, our findings indicate that profitability remains a potential barrier in the sustainment phase. This suggests that any changes in customer purchasing behaviour or other factors influencing financial viability (e.g., cost of healthy food) may lead to discontinuation of healthy food retail practices. However, a recent systematic review of costs associated with healthy food retail interventions in foodservice contexts suggests that such interventions typically result in neutral or favourable financial outcomes, highlighting a potential mismatch between perceived and actual financial outcomes [53].

Our findings suggest that healthy food and drink supplier availability may be a key systems-level determinant of sustainment, emerging as one of the most frequently reported and highly ranked enablers and barriers among foodservice operators. Addressing the availability and accessibility of healthier items through supplier and manufacturer engagement could have broad influence across foodservice contexts, given that suppliers operate across multiple settings. Future research should examine suppliers’ barriers to providing healthier products and identify strategies to improve the supply of healthier options. Although supplier availability was commonly reported as a determinant by foodservice operators in this study, support with finding new suppliers was not frequently ranked as helpful. This indicates that working with existing suppliers to identify and expand their healthier product ranges may be more effective than helping foodservice operators identify new suppliers, given the potential practical challenges related changing suppliers.

Foodservice operators highlighted a clear obligation to provide healthier options, and practitioners emphasised leadership support as a key enabler to sustainment. These findings indicate that organisational leadership within governing organisations (e.g., healthcare facilities or local governments) plays an important role in sustaining healthy food retail practices. Similar findings have been reported in Australian university vending settings [51] and New Zealand healthcare organisations [34], where organisational leadership has been identified as a key determinant of sustainment. The importance of organisational leadership for sustainment is also well established in the implementation science literature, including in the Integrated Sustainability Framework [47]. Leadership is also reflected in the sustainment-explicit ERIC framework, which includes several strategies involving organisational leaders (e.g. “involve executive boards”, “mandate change”) [26]. In public health settings, governing organisations are often expected to lead or oversee the sustainment of healthy food retail practices within their food outlets [19], and leadership can play a key role in organising ongoing sustainment support (e.g., compliance monitoring) [38]. Organisational leaders therefore represent important actors to engage when designing healthy food retail interventions. A “clear obligation” may also be interpreted as formal health directives or community expectations for health-promoting settings, such as schools and healthcare organisations that are expected to lead health-promoting practices [15, 19], highlighting the importance of government and community support for implementation and long-term sustainment [54].

### Implications for practice and policy

Foodservice operators in this study prioritised supports that can help maintain financial viability and prepare food-outlet level champions to lead change. This reinforces the need for business-oriented supports within healthy food retail programs, such as assistance to food outlets with developing a business case for healthy food [27]. While the ERIC glossary identifies a range of financial strategies relevant to sustainment of health interventions (e.g., “Access new funding”, “Develop disincentives”) [26], the emphasis on business-focused support appears particularly relevant in the food retail context. Previous research has proposed approaches to adapting ERIC strategies for community settings, but these adaptations primarily focus on implementation and include limited consideration of business-focused strategies, reflecting the broader range of community settings included in these articles [55, 56]. Our findings suggest that further research is needed to develop sustainment-explicit strategies that acknowledge and address the business context of food retail settings.

This study also provides novel insights by suggesting that healthy food availability may be more feasible to sustain compared to reducing the range of less healthy products or the use of pricing strategies. Difficulties in maintaining reductions in less healthy products may be partly attributable to customer complaints [33]. While limited research has examined the long-term sustainability of pricing interventions, existing evidence indicates that many healthier foods and drinks are often unaffordable for consumers [57], which may contribute to difficulties in sustaining pricing strategies in Australian foodservice settings. These findings indicate that pricing and product-restriction strategies likely require stronger policy and implementation supports rather than solely relying on local implementors. For example, Backholer et al. (2019) [58] highlight the importance of policies that reduce the prevalence and influence of unhealthy food and drink price promotions as part of a comprehensive approach to improving population diets (e.g., legislation to restrict price promotions on unhealthy food and drinks).

Policy frameworks may also need stronger monitoring and accountability mechanisms. Current monitoring systems tend to focus primarily on product availability [15, 19], and a broader range of healthy food retail practices (beyond product availability monitoring) may require ongoing oversight to support sustainment. Monitoring systems also vary across Australian jurisdictions in scope and frequency, which may lead to differences in enforcement and contribute to inequities in access to healthy food in different settings [19]. Strengthening and harmonising monitoring structures across states and territories may therefore support more consistent sustainment of healthy food retail practices.

While this study was conducted in Australia, the findings may be relevant to countries with similar policy and foodservice contexts (e.g. New Zealand and Canada), and the survey could be adapted for application in other settings.

### Strengths and limitations

Key strengths of this study included the co-design of the research survey with representatives from Australian Departments of Health and state health promotion agencies responsible for healthy food retail policies and/or implementation support, enhancing its policy and practice relevance, and the use of established implementation science frameworks to inform survey design. Additional strengths include the national sample, inclusion of both foodservice operators and practitioners, and coverage of diverse foodservice settings.

Some limitations should also be noted. Given the exploratory nature of this study and the limited evidence base on sustainment of healthy food retail interventions, survey items were informed by theory and existing literature rather than validated instruments. The use of non-probability sampling may limit generalisability, and smaller subgroup sizes reduced the strength of comparative analyses. Another limitation is that this study relied on self-reported sustainment without objective verification, and the sustainment timeframe was not defined in the survey. Finally, not all potential determinants of sustainment were included as survey items to minimise respondent burden; instead, the survey prioritised most common determinants identified in our previous systematic review [49] and other relevant evidence [19, 25, 48]. Future research could therefore focus on more in-depth exploration of sustainment determinants and support strategies, including through longitudinal designs to compare perceived and observed sustainment over time.

## Conclusion

Sustaining healthy food retail practices over time can be challenging, with this study suggesting that pricing strategies and product-restriction practices are particularly difficult to maintain. Key determinants across foodservice settings that support or hinder sustainment relate to supplier access, financial viability, and leadership support. These findings highlight the need for higher-level support that involves multiple key actors across the food retail system to ensure the long-term sustainment of healthy food retail practices. Failure to adequately support sustainment may discourage foodservice operators from re-engaging with healthy food retail initiatives following unsuccessful attempts, limiting longer-term population health impact.

## Supplementary Information

Supplementary Material 1.

Supplementary Material 2.

Supplementary Material 3.

## Data Availability

The datasets used and/or analysed during the current study are available from the corresponding author on reasonable request.

## Abbreviations

ERIC: Expert Recommendations for Implementing Change (a taxonomy of implementation and sustainment strategies)
IRSD: Index of Relative Socioeconomic Disadvantage
ISF: Integrated Sustainability Framework
MD: Mean difference
SEIFA: Socio-Economic Indexes for Areas
