# Supplementary material for: Foodservice operator and implementation support practitioner needs for sustaining healthy food retail practices in Australian foodservice settings: findings from a national cross-sectional survey
Source: Int J Behav Nutr Phys Act. 2026 Aug 5;23:106. doi: 10.1186/s12966-026-01944-7 (PMC13729286; doi:10.1186/s12966-026-01944-7)
Supplement: Supplementary file 1 [file 12966_2026_1944_MOESM1_ESM.docx]

**Supplementary File 1. Survey instrument used in the Healthy Food Retail Sustainment study**

Questions are shown verbatim. Display and branching logic indicate which questions were shown to which participants. Only items relevant to the present analysis are marked as included. Questions used single-response options unless otherwise indicated (e.g., “select all that apply”).

| Digital Healthy Food Retail Implementation tool survey | | | | | | |
| --- | --- | --- | --- | --- | --- | --- |
| # | Audience (retailers^[1]^ or practitioners) | Question | Response options | Construct | Display and branching logic (if applicable) | Item included in the analysis |
| ELIGIBILITY AND SCREENING | | | | | | |
| 1 | All | Are you based in Australia, aged over 18 years, able to provide informed consent, and are able to read, speak and understand English? | Yes; No | Eligibility screening | Screen out if “No” | No (just for screening purposes) |
| 2 | Practitioners and retailers (state distributed survey) | Do you have experience in trying to make healthier changes in Australian retail food service outlets that mainly sells food for immediate consumption (e.g. a café, kiosk, or canteen)? | - Yes, as a retail outlet owner, manager, employee or volunteer - Yes, in a role (e.g., health promotion, environmental health officer, nutrition co-ordinator, nutritionist, dietitian, or other role) supporting food service retailers - No |  | Screen out if “No”  “Yes”, assigns participant to retailer or practitioner pathways |  |
|  | Retailers (Qualtrics panel) | Do you **own, manage, work or volunteer** in a food outlet that mainly sells food for immediate consumption (e.g. a café, kiosk, or canteen)? | Yes; No |  | Screen out if “No” |  |
| 3 | All retailers | Is your food outlet part of a fast food chain (such as a franchise or brand) that has more than 20 locations? | Yes; No |  | Screen out if “Yes” |  |
| 4 | All | We are pleased to confirm you are eligible to participate. **The survey will take approximately 15 minutes. Your involvement in the study is anonymous and voluntary.** If you choose to provide your email address to be contacted by the researchers or to go into a prize draw, these details will not be linked to the survey responses you provide. **Before agreeing to participate, please review the Plain Language Statement below** for detailed information about this research project. Click the link below to access the statement, and you may also download a copy for your records. Plain language statement. By clicking "Yes," you are acknowledging that you have read the plain-language statement and agree to participate in this survey. | - Yes, I have read the plain language statement and agree to participate in this research - No, I do NOT agree to participate in this research | Consent | Screen out if “No” | No (consent) |
| DEMOGRAPHICS AND CONTEXT | | | | | | |
| 5 | Practitioners | Which of the following best describes your role? | - Health promotion officer - Environmental health officer - Nutrition co-ordinator - Nutritionist - Dietitian - Other (please specify) | Participant role | Relevant question displayed to relevant participant group | Yes |
|  | All retailers |  | - Food outlet owner - Manager - Shift supervisor - Staff member (customer facing role) - Staff member (food preparation/ kitchen) - Volunteer - Other (please specify) |  |  |  |
| 6 | Practitioners | Who employed you to support retailers? | - Local government - Local public health unit - State government - Non-government organisation - Contracted as a private consultant - Employed directly by the retail business - Hospital or health service - Other (please specify) |  | Displayed to practitioners only | No |
| 7 | Practitioners | How long have you been supporting retailers to implement healthier food and drink initiatives? | - Less than 2 years - 2-4 years - 4-6 years - More than 6 years | Work experience | Relevant question displayed to relevant participant group | No |
|  | All retailers | How long have you been in your current role? |  |  |  |  |
| 8 | All | Which state or territory do you mainly work in? | - Australian Capital Territory - New South Wales - Northern Territory - Queensland - South Australia - Tasmania - Victoria - Western Australia | Location | N/A | Yes |
| 9 | Practitioners | Please enter either the postcode(s) or the name of the local government area (LGA) you most frequently support food retail outlets in. | 4 x open text box options for post code (numeric responses only)  1 x open text box option for local government area | Location (used to derive SEIFA IRSD) | Relevant question displayed to relevant participant group | Yes |
|  | All retailers | Please enter either the name of the local government area (LGA) or postcode(s) of the outlets you own, manage or work in |  |  |  |  |
| 10 | Practitioners | What retail settings do you support? Select all that apply. | - Schools (primary or secondary) - Hospital and health service facility retail (including charitable operations) - Sports and recreation setting retail (e.g. aquatic centre, stadium) - Sports clubs - Arts, cultural or events setting retail (e.g. theatre, library, community/arts centres) - Workplace setting retail - Park setting retail - University setting retail - Other (please specify) | Food retail setting | Relevant question displayed to relevant participant group | Yes |
|  | All retailers | Which best describes your food outlet’s/outlets' **setting(s)**? Select all that apply. | - School (primary or secondary) - Hospital and health service facility retail (including charitable operations) - Sports and recreation setting retail (e.g. aquatic centre, stadium) - Sports clubs (e.g. netball club) - Arts, cultural or events setting retail (e.g. theatre, library, community/arts centres) - Workplaces - Parks - Universities - Private setting (none of the above) - Other (please specify) |  |  |  |
| HEALTHY FOOD RETAIL EXPERIENCE | | | | | | |
| *Throughout this survey we use the terms 'healthier' and 'less healthy'. These terms should be interpreted in relation to your state or territory's specific nutrition guidelines or classification system for the setting(s) you work in. These are generally in line with the Australian Dietary Guidelines and promote the intake of fruits and vegetables, lean proteins and wholegrains, while limiting the intake of foods high in saturated fat, added salt or sugars, and alcohol. The survey will ask you about your experience in helping to make healthier changes (i.e., through changes to product, price, placement and/or promotion of items) in food service outlets.* | | | | | | |
| 11 | Retailers (Qualtrics panel) | Do you have **experience** making or attempting to make changes in your food outlet to encourage customers to choose healthier foods or drinks? | - Yes, I have experience - No, but we are interested in encouraging customers to choose healthier options - No, and we are not currently interested in encouraging customers to choose healthier options | Healthy food retail experience | Displayed to retailers (Qualtrics panel) only  Assigns participant to ‘experienced retailer’, ‘potential (healthy food) retailer’ and ‘not interested retailer’ pathways | Yes |
| 12.1 | Practitioners | What types of changes have you helped food outlets with to encourage customers to buy healthier food and/or drink options? Select all that apply. | Response options for each item:   - Not attempted - Attempted but no longer in place - Achieved recently - Achieved and kept in place over time   Items:   - The range of “healthier” food or drink products was increased - The range of “less healthy” foods or drinks was reduced - Recipes or food preparation methods changed to improve the nutritional profile of items - Placement strategies to make healthier options more prominent (e.g. placing healthier items at eye level or near registers) - Pricing strategies to encourage healthier purchases (e.g. pricing discounts on healthier items, increasing prices of less healthy options) - New promotional materials, messages, or activities (e.g. labelling, signage) were introduced to promote healthier options - Promotional materials or activities on less healthy options were removed (e.g. branded displays or signage promoting less healthy items) | Experience with healthy food retail practices | Displayed to practitioners and experienced retailers only  Relevant question displayed to relevant participant group | Yes |
|  | Experienced retailers | Which of the following changes has your food outlet made to encourage customers to buy healthier food and/or drink options? |  |  |  |  |
| 12.2 | All | Is there another type of change that wasn't included in the above question? | Open text box with response options   - Attempted but no longer in place - Achieved recently - Achieved and kept in place over time |  | Displayed to practitioners and experienced retailers only | Yes |
| 13 | Practitioners | In trying to make these changes, what types of **support** have you provided? Select all that apply. | - Conducting an audit or spot check of the healthiness (e.g. available products, pricing, placement, and promotion) of food and drinks - Identifying new suppliers - Identifying new or alternative healthier products - Labelling (e.g. traffic light labelling sheets ‘everyday’ or ‘occasional’ labelling, help or line in a nutrition or naming of menu items) - Getting feedback from customers - Engaging with other retailers who have successfully made their outlets healthier - Small changes to test and refine implementation strategies - Identifying someone to champion healthier changes in the food outlet - Developing an action plan or retailer policy - Advice on recipe or menu changes - Not sure/none - Other (please specify) | Implementation and sustainment support | Not displayed to retailers | No  (reported elsewhere) |
| 14 | Practitioners | What additional **resourcing** have you arranged for your retailer? Select all that apply. | - Funding for equipment or outlet updates that make it easier to prepare or sell healthier items - Staff training on topics other than classifying items - Student placement through a local university or a state/territory health agency - Staff training on classifying food and drink products and/or conducting audits - Not sure/none - Other (please specify) | Implementation and sustainment resourcing | Not displayed to retailers | No  (reported elsewhere) |
| DETERMINANTS ^[2]^ | | | | | | |
| - | Experienced retailers | **Next, we’d like you to think about the factors that have influenced your ability to make and keep healthy changes in place in your food outlet.** Healthy changes refer to any changes you’ve made to your product offerings or practices to support customers in choosing healthier food and drink options - as described in the previous question. | | | Not displayed to practitioners | - |
| - | Potential and not interested retailers | **Next, we’d like you to think about the factors that may influence your ability to make and keep healthy changes in place in your food outlet.** Healthy changes refer to any changes you’ve made to your product offerings or practices to support customers in choosing healthier food and drink options - as described in the previous question. | | |  |  |
| 15.1 | Practitioners | Reflecting on your past experiences, what has **helped** in supporting outlets to make healthier changes? For each supporting factor that you have experienced, select whether it **helped when initially making healthier changes**, **helped in keeping healthier changes in place over time**, **both**, or **neither/ not sure**. | For each supporting factor that you have experienced, please indicate whether it:   - Helped when initially making healthier changes - Helped when keeping healthier changes in place over time - Both - Neither / not sure   Items: | Enablers to implementation and sustainment^[3]^: | Relevant question displayed to relevant participant group | Yes |
|  |  |  | Dedicated technical support (e.g., someone who can provide advice on why an item is classified as it is, and suggest potential alternatives) | Technical assistance, support and supervision |  |  |
|  |  |  | Availability of useful resources (e.g., checklists, guidelines, planograms, pre-tested recipe ideas) | Material resources / Technical support |  |  |
|  |  |  | Liaising with suppliers to identify healthier items | Food supply and supplier-related factors |  |  |
|  |  |  | Strong rapport between retailers and those supporting them to make change | Practitioner – retailer partnership / Partnership and engagement |  |  |
|  |  |  | Staged implementation strategies (e.g., adding healthier items incrementally or before removing less healthy items) | Trialability / Adaptation |  |  |
|  |  |  | Support from state/territory funded agencies or peak bodies | External partners, support and resources / Governmental leadership |  |  |
|  |  |  | Support and advice from other practitioners | Peer support / Partnership and engagement |  |  |
|  |  |  | My previous experience with supporting retailers to make changes | Practitioner knowledge, skills and expertise |  |  |
|  |  |  | Providing retailers with resources | Material resources / Technical support |  |  |
|  |  |  | Support from the leadership/executive/board in the food outlet's parent organisation | Organisational leadership |  |  |
|  | Experienced retailers | Reflecting on your past experiences, what has **helped** you make healthy changes? For each supporting factor that you have experienced, select whether it   - helped with **initially making healthy changes** - helped **keeping healthy changes in place** - or both | For each factor, please indicate whether it:   - Would help when initially making healthy changes - Would help keep changes in place over time - Both - Not sure / not relevant   Items: | Enablers to implementation and sustainment^[4]^: |  |  |
|  | Potential and not interested retailers | Which of the following factors do you think would make it easier to make and keep healthy changes in food outlets? For each supporting factor that you have experienced, select whether it   - would help with **initially making healthy changes** - would help **keep healthy changes in place** - or both | Dedicated technical support (e.g., someone who can provide advice on why an item is classified as it is, and suggest potential alternatives) | Technical assistance, support and supervision |  |  |
|  |  |  | Availability of useful resources (e.g., checklists, guidelines, planograms, pre-tested recipe ideas) | Material resources / Technical support |  |  |
|  |  |  | Healthy food and supplier availability | Food supply and supplier-related factors |  |  |
|  |  |  | My food outlet has a clear obligation to provide healthy food and drink options | Organisational leadership, mission and policies / Governmental leadership |  |  |
|  |  |  | Staged implementation strategies (e.g., adding healthier items incrementally or before removing less healthy items) | Trialability / Adaptation |  |  |
|  |  |  | Customers purchase or request healthy options | Customer purchasing behaviour |  |  |
|  |  |  | Monitoring and evaluation of healthy changes made | Fidelity, monitoring and enforcement |  |  |
|  |  |  | My food outlet manager’s and/or staff’s skills and knowledge | Food outlet staff knowledge, skills and expertise |  |  |
|  |  |  | My and/or my food outlet’s previous experience making healthy changes | Food outlet manager’s or staff’s previous experience ^[5]^ |  |  |
|  |  |  | Food outlet manager’s and/or staff’s willingness or motivation to make healthy changes | Retailer or staff characteristics, motivation and attitudes |  |  |
| 15.2 | Practitioners | Was there anything else that supported outlets to make healthier changes that wasn't listed in the above options? | Open text box with options:   - Helped when initially making changes - Helped to keep changes in place over time | Enablers to implementation and sustainment | Relevant question displayed to relevant participant group |  |
|  | Experienced retailers | Was there anything else that has supported you to make healthier changes that wasn't listed in the above options? |  |  |  |  |
|  | Potential and not interested retailers | Was there anything else that may support you to make healthier changes that wasn't listed in the above options? |  |  |  |  |
| 16 | Practitioners | Thinking about these factors that have helped you in supporting retailers **making healthier changes**, drag and drop up to 10 factors to **rank** by importance, with 1 as the most important. | Drag and drop up to 10 options (‘Helped when initially making changes‘ options selected in Q15) and ranking them | Key enablers to implementation | Relevant question displayed to relevant participant group | No  (reported elsewhere) |
|  | All retailers | Thinking about these factors that are helpful when **initially making healthier changes**, drag and drop these to rank up to 10 factors in order of importance, with 1 as the most important. |  |  |  |  |
| 17 | Practitioners | Thinking about these factors that have helped you in supporting retailers **keep healthier changes in place** over time, drag and drop up to 10 factors to **rank** by importance, with 1 as the most important. | Drag and drop up to 10 options (‘Helped to keep changes in place over time’ options selected in Q15) and ranking them | Key enablers to sustainment | Relevant question displayed to relevant participant group | Yes |
|  | All retailers | Thinking about these factors that are helpful when **keeping healthier changes in place** over time, drag and drop up to 10 factors in order of importance, with 1 as the most important. |  |  |  |  |
| 18.1 | Practitioners | Reflecting on your past experiences, what has made it **harder** to support outlets to make healthier changes? For each that you have experienced, select whether it **made it harder in initially making healthier changes**, **made it harder to keep healthier changes in place over time**, **both**, or **neither/ not sure** | For each factor that you have experienced, please indicate whether it:   - Made it harder when initially making healthier changes - Made it harder to keep changes in place over time - Both - Neither / not sure   Items: | Barriers to implementation and sustainment^[6]^: | Relevant question displayed to relevant participant group | Yes |
|  |  |  | High staff turnover within the retail setting | Organisational stability / staffing turnover |  |  |
|  |  |  | Lack of rapport within retailers | Practitioner – retailer partnership / Partnership and engagement |  |  |
|  |  |  | Retailers feel implementation is a prescriptive instead of a collaborative process | Partnership and engagement |  |  |
|  |  |  | Retailers concerned about losing customers or money | Profitability and cost / Customer purchasing behaviour |  |  |
|  |  |  | Difficulty accessing healthier options or ingredients from suppliers | Food supply and supplier-related factors |  |  |
|  |  |  | Lack of support or communication from the policy setter | Communication / Governmental leadership |  |  |
|  |  |  | Retailer concern about increased workload | Burden and complexity |  |  |
|  |  |  | Retailers not having the skills or knowledge to implement changes | Retailer and/or food outlet staff knowledge, skills and expertise |  |  |
|  |  |  | Lack of access to supportive or practical resources | Material resources / Technical support |  |  |
|  |  |  | Not enough time/capacity/FTE in my current role | Practitioner capacity |  |  |
|  |  |  | Unclear or open to interpretation what counts as a healthier change | Complexity / Practitioner knowledge, skills and expertise |  |  |
|  |  |  | Not sure where to start or what to do next | Practitioner knowledge, skills and expertise |  |  |
|  | Experienced retailers | Reflecting on your past experiences, what has made it **harder** to make healthier changes?  For each factor, select whether it makes it harder to   - **initially make healthier changes** - **keep healthier changes in place** - both - or not sure/not relevant | For each factor, please indicate whether it:   - Would make it harder when initially making healthy changes - Would make it harder to keep changes in place over time - Both - Not sure / not relevant   Items: | Barriers to implementation and sustainment^[7]^: |  |  |
|  | Potential and not interested retailers | Which of the following factors do you think would make it **harder** to make and keep healthy changes in food outlets?  For each factor, select whether it makes it harder to   - **initially make healthier changes** - **keep healthier changes in place** - both - or not sure/not relevant | High food outlet staff turnover | Organisational stability / staffing turnover |  |  |
|  |  |  | Competition between food outlets (e.g., other outlets keep stocking less healthy items or healthier items at lower prices) | Competition |  |  |
|  |  |  | Limited access to practical resources (e.g., implementation support tools, checklists, guidelines, pre-tested recipe ideas) | Material resources / Technical support |  |  |
|  |  |  | Customer demand for less healthy options (e.g., confectionery) | Customer purchasing behaviour |  |  |
|  |  |  | There’s no clear expectation for my outlet to provide healthier food and drinks | Organisational leadership, mission and policies / Governmental leadership |  |  |
|  |  |  | Difficulty accessing healthier options or ingredients from suppliers | Food supply and supplier-related factors |  |  |
|  |  |  | Healthy changes have complex or strict rules that must be followed, with little room to adapt them to my outlet | Burden and complexity / Adaptability |  |  |
|  |  |  | My food outlet, manager and/or staff are resistant to making healthy changes | Retailer or staff characteristics, motivation and attitudes |  |  |
|  |  |  | Additional costs and/or profit losses associated with healthy changes | Profitability and cost |  |  |
|  |  |  | Lack of access to dedicated support (from individuals or organisations) | Technical assistance, support and supervision |  |  |
|  |  |  | Burden and/or additional workload associated with healthy changes | Burden and complexity |  |  |
|  |  |  | Unclear or open to interpretation what counts as a healthy change | Complexity / Retailer and/or food outlet staff knowledge, skills and expertise |  |  |
|  |  |  | Not sure where to start or what to do next | Retailer and/or food outlet staff knowledge, skills and expertise |  |  |
|  |  |  | Lack of compliance monitoring or incentives | Fidelity, monitoring and enforcement |  |  |
| 18.2 | Practitioners | Was there anything else that made it harder to support outlets to make healthier changes that wasn't listed in the above options? | Open text box with options:   - Made it harder when initially making changes - Made it harder when keep changes in place over time | Barriers to implementation and sustainment | Relevant question displayed to relevant participant group | Yes |
|  | All retailers | Was there anything else that can make it harder to make healthier changes that wasn't listed in the above options? |  |  |  |  |
| 19 | Practitioners | Thinking about these things that have made it harder to support retailers **to initially make healthier changes,** drag and drop up to 10 factors to **rank** by importance, with 1 as the most important. | - Drag and drop up to 10 options (‘Made it harder when initially making changes ‘options selected in Q18) and ranking them | Key barriers to implementation | Relevant question displayed to relevant participant group | No  (reported elsewhere) |
|  | All retailers | Thinking about these things that can make it **harder to initially make healthier changes,** drag and drop up to 10 factors in order of importance, with 1 as the most important. |  |  |  |  |
| 20 | Practitioners | Thinking about these **things that have made it harder to support retailers to keep healthier changes in place** over time, drag and drop these (up to 10) to **rank** them by importance, with 1 as the most important. | - Drag and drop up to 10 options (‘Made it harder when keep changes in place over time’ options selected in Q18) and ranking them | Key barriers to sustainment | Relevant question displayed to relevant participant group | Yes |
|  | All retailers | Thinking about these things that can make it **harder to keep healthier changes in place** over time, drag and drop up to 10 factors in order of importance, with 1 as the most important. |  |  |  |  |
| SUPPORT (RETAILERS) ^[8]^ | | | | | | |
| - | Experienced and potential retailers | Next, we’d like you to think about any support you would like to access to help you make and keep healthy changes in place in your outlet. | | | Not displayed to practitioners or not interested retailers | - |
| 21 | Experienced and potential retailers | Please select any support related to ‘**Relationships and partners’** that you would like to access. Select all that apply. | Items: | Retailer implementation and sustainment support needs^[9]^: | Not displayed to practitioners or not interested retailers | Yes (through the ranking Q only) |
|  |  |  | Feedback on how my food outlet is performing against nutrition/food policy targets | Audit and provide feedback |  |  |
|  |  |  | Support to plan healthy changes in a way that works for my outlet | Tailor strategies |  |  |
|  |  |  | Help auditing the healthiness of our food environment, product offerings, or compliance with policy | Audit and provide feedback / Purposely reexamine the implementation |  |  |
|  |  |  | Support with getting feedback from customers | Obtain and use consumer feedback |  |  |
|  |  |  | Engaging customers in healthy options | Involve consumers |  |  |
|  |  |  | Help identifying and training a staff member to lead healthy changes in my outlet | Identify and prepare champions |  |  |
|  |  |  | Other (please specify)  Not sure/none |  |  |  |
| 22 | Experienced and potential retailers | Please select any support related to ‘**Making changes’** that you would like to access. Select all that apply. | Items: | Retailer implementation and sustainment support needs^[10]^: | Not displayed to practitioners or not interested retailers | Yes (through the ranking Q only) |
|  |  |  | Knowing where to start | Develop a formal implementation blueprint |  |  |
|  |  |  | Access to external funding or financial incentives to support healthy changes | Access new funding |  |  |
|  |  |  | Support identifying new suppliers | Facilitation / Provide local technical assistance |  |  |
|  |  |  | Support identifying new or alternative healthy products | Facilitation / Provide local technical assistance |  |  |
|  |  |  | Advice or support on pricing, store display, healthy foods and drinks | Provide local technical assistance |  |  |
|  |  |  | Advice or support on labelling (e.g. traffic light labelling sheets ‘everyday’ or ‘occasional’ labelling, help or line in a nutrition or naming of menu items) | Provide local technical assistance / Use an implementation advisor |  |  |
|  |  |  | Improving the skills of the staff to prepare healthier options | Conduct educational meetings |  |  |
|  |  |  | Support or advice on small changes I can try and adjust to suit my food outlet | Conduct cyclical small tests of change / Stage implementation scale up / Promote adaptability |  |  |
|  |  |  | Recipe modifications to deal with food allergies and intolerance while meeting nutrition standards | Provide local technical assistance / Use an implementation advisor |  |  |
|  |  |  | Other (please specify)  Not sure/none |  |  |  |
| 23 | Experienced and potential retailers | Please select any support related to '**Factors influencing business outcomes**' that you would like to access. Select all that apply. | Items: | Retailer implementation and sustainment support needs^[11]^: | Not displayed to practitioners or not interested retailers | Yes (through the ranking Q24 only) |
|  |  |  | Advice on planograms or stock display | Change physical structure and equipment |  |  |
|  |  |  | Advice on setting the everyday price of items | Alter consumer fees |  |  |
|  |  |  | Advice on price promotions or special deals | Alter consumer fees / Increase demand |  |  |
|  |  |  | Making money by selling healthy options | Increase demand |  |  |
|  |  |  | Advice on food or financial waste management | Use an implementation advisor / Facilitate relay of data (e.g. sales) to retailers |  |  |
|  |  |  | Support to make the food outlet more environmentally sustainable | Use an implementation advisor /  Tailor strategies |  |  |
|  |  |  | Resources to promote the healthy food options to customers | Increase demand |  |  |
|  |  |  | Advice on marketing or advertising for the food outlet to customers | Increase demand / Use mass media |  |  |
|  |  |  | Other (please specify)  Not sure/none |  |  |  |
| 24 | Experienced and potential retailers | Thinking about these support options, please indicate which ones you think would help your food outlet **keep healthier changes in place** over time. Drag and drop up to 5 options in order of helpfulness, with 1 being the most helpful. | - Drag and drop up to 5 options (‘Made it harder when keep changes in place over time’ options selected in Q21-23) and ranking them | Sustainment support needs | Not displayed to practitioners or not interested retailers | Yes |
| 25 | Experienced retailers | Where has your outlet received practical or planning support from **in the past** to help make and/or keep healthy changes in place in your outlet? *Select all that apply* | - I have not previously received support - Internal organisational support (e.g. dietitian for hospital-based retailer) - Local government - Private consultants (e.g. nutritionists) - State-based support service (e.g. Healthy Eating Advisory Service) - NGOs (e.g. Cancer Council) - Retail associations - Food distributors/suppliers - Universities / academic institutions - Other (please specify) | Sustainment support experience | Not displayed to practitioners or non-experienced retailers | No (reported elsewhere) |
| RESOURCES | | | | | | |
| *Questions related to participant experiences with healthy food retail resources not relevant to this study (reported elsewhere)* | | | | | | |
| CLOSING | | | | | | |
| - | All retailers | **You're nearly done!**  Just a few final questions to help us better understand your food outlet's unique context. | | | Not displayed to practitioners |  |
| 26 | All retailers | On average, how many staff (including yourself, all full-time, part-time, casual staff, and volunteers) work during a typical shift at your food outlet? | - 1–2 staff - 3–5 staff - 6–10 staff - 11–20 staff - More than 20 staff | Outlet size | Not displayed to practitioners |  |
| 27 | All retailers | What types of staff work at your food outlet? *Please select all that apply* | - Full-time staff - Part-time staff - Casual staff - Volunteers - Other (please specify) | Outlet type (staffing) | Not displayed to practitioners |  |
| 28 | All retailers | What types of food does your food outlet(s) mainly offer? *Please select all that apply* | - Commercially packaged snacks (e.g. confectionery, nut mixes, cereal products purchased from suppliers and sold as-is) - Commercially prepared meals (e.g. pre-packaged sandwiches, wraps, pies, hot foods purchased from suppliers) - Commercially packaged drinks (e.g. bottled or canned soft drinks, juices, flavoured milk) - Alcoholic drinks (alcoholic, from tap, mixed drinks) - Made-to-order food (e.g. eggs on toast, burgers, salads—freshly prepared upon customer request) - Made-to-order drinks (e.g. coffee, smoothies, milkshakes—freshly prepared upon customer request) - Ready-to-eat foods prepared in-house (e.g. sandwiches, wraps, salads, cakes, hot food in bain-marie—prepared on-site but sold ready for quick selection) | Outlet type (offerings) | Not displayed to practitioners |  |
| 29 | All | Is there anything else you’d like to share about your experience with healthy food retail initiatives? | Open text entry | - | - | No |

**References**

1.	Shelton RC, Cooper BR, Stirman SW. The Sustainability of Evidence-Based Interventions and Practices in Public Health and Health Care. Annual Review of Public Health. 2018;39(1):55-76; doi: 10.1146/annurev-publhealth-040617-014731.

2.	Boelsen-Robinson T, Blake MR, Brown AD, Huse O, Palermo C, George NA, et al. Mapping factors associated with a successful shift towards healthier food retail in community-based organisations: a systems approach. Food Policy. 2021;101; doi: 10.1016/j.foodpol.2021.102032.

3.	Nathan N, Powell BJ, Shelton RC, Laur CV, Wolfenden L, Hailemariam M, et al. Do the Expert Recommendations for Implementing Change (ERIC) strategies adequately address sustainment? Frontiers in Health Services. 2022;2; doi: 10.3389/frhs.2022.905909.

1. Retailer = foodservice operator (e.g., foodservice outlet owner, manager, staff or volunteer)
2. Question wordings vary slightly by audience and participant experience to reflect past experience versus perceived relevance.
3. Item-level constructs are mapped to determinants within the Integrated Sustainability Framework [1], with adaptations for food retail contexts
4. Item-level constructs are mapped to determinants within the Integrated Sustainability Framework [1], with adaptations for food retail contexts.
5. This determinant was informed by the Systems Thinking Approach for Retail Transformation (START) framework [2].
6. Item-level constructs are mapped to determinants within the Integrated Sustainability Framework [1], with adaptations for food retail contexts
7. Item-level constructs are mapped to determinants within the Integrated Sustainability Framework [1], with adaptations for food retail contexts
8. These questions were displayed only to retailers with experience or interest in implementing healthy food retail practices.
9. Item-level constructs are mapped to strategies within the sustainment-explicit Expert Recommendations for Implementing Change [3], with adaptations for food retail contexts
10. Item-level constructs are mapped to strategies within the sustainment-explicit Expert Recommendations for Implementing Change [3], with adaptations for food retail contexts
11. Item-level constructs are mapped to strategies within the sustainment-explicit Expert Recommendations for Implementing Change [3], with adaptations for food retail contexts
