# Supplementary material for: Foodservice operator and implementation support practitioner needs for sustaining healthy food retail practices in Australian foodservice settings: findings from a national cross-sectional survey
Source: Int J Behav Nutr Phys Act. 2026 Aug 5;23:106. doi: 10.1186/s12966-026-01944-7 (PMC13729286; doi:10.1186/s12966-026-01944-7)
Supplement: Supplementary file 2 [file 12966_2026_1944_MOESM2_ESM.docx]

**Additional File 2:** Differences in determinants of sustained healthy food retail practices selected by foodservice operators with and without healthy food retail experience

| DETERMINANT | % selecting determinant (95% CI) ^[1]^ | Selection difference  (mean % difference, 95% CI) |
| --- | --- | --- |
| ENABLERS | n=211 | No experience (n=63) v. experienced (n=157) ^[2]^ |
| Monitoring and evaluation of healthy changes made | 72 (66, 78) | -3 (-2, 10) |
| Healthy food and supplier availability | 69 (63, 75) | -6 (-19, 8) |
| My food outlet has a clear obligation to provide healthy food and drink options | 66 (60, 72) | +5 (-9, 19) |
| Food outlet manager's and/or staff's willingness or motivation to make healthy changes | 66 (59, 72) | **+14 (1, 28) *** |
| Customers purchase or request healthy options | 63 (56, 69) | -6 (-20, 9) |
| My food outlet manager's and/or staff's skills and knowledge | 62 (56, 68) | -3 (-17, 12) |
| My and/or my food outlet's previous experience making healthy changes | 57 (51, 64) | +4 (-10, 19) |
| Staged implementation strategies  (e.g., adding healthier items incrementally or before removing less healthy items) | 55 (49, 62) | +5 (-10,19) |
| Availability of useful resources  (e.g., checklists, guidelines, planograms, pre-tested recipe ideas) | 55 (48, 61) | **-18 (-33, -4) *** |
| Dedicated technical support (e.g., someone who can provide advice on why an item is classified as it is, and suggest potential alternatives) | 50 (43, 57) | +3 (-11, 18) |
| BARRIERS | n=211 | No experience (n=62) v. experienced (n=149) ^[3]^ |
| Additional costs and/or profit losses associated with healthy changes | 59 (52, 65) | -6 (-20, 9) |
| My food outlet, manager and/or staff are resistant to making healthy changes | 59 (52, 65) | -6 (-20, 9) |
| Burden and/or additional workload associated with healthy changes | 57 (51, 64) | +3 (-12, 18) |
| Difficulty accessing healthier options or ingredients from suppliers | 55 (49, 62) | -1 (-15, 14) |
| Lack of access to dedicated support (from individuals or organisations) | 55 (49, 62) | +4 (-11, 19) |
| Limited access to practical resources (e.g., implementation support tools, checklists, guidelines, pre-tested recipe ideas) | 55 (48, 61) | -4 (-19, 11) |
| Lack of compliance monitoring or incentives | 54 (47, 61) | -10 (-25, 5) |
| Not sure what to do next | 53 (46, 60) | 0 (-15, 15) |
| Customer demand for less healthy options (e.g., confectionery) | 49 (42, 56) | -3 (-18, 12) |
| Competition between food outlets (e.g., other outlets keep stocking less healthy items or healthier items at lower prices) | 48 (42, 55) | +14 (-1, 29) |
| There’s no clear expectation for my outlet to provide healthier food and drinks | 47 (41, 54) | +4 (-11, 17) |
| Unclear or open to interpretation what counts as a healthy change | 45 (38, 52) | +7 (-8, 22) |
| Healthy changes have complex or strict rules that must be followed, with little room to adapt them to my outlet | 42 (36, 49) | 0 (-15, 14) |
| High food outlet staff turnover | 37 (31, 44) | -7 (-20,8) |

*This table presents determinants (enablers and barriers) of sustained healthy food retail practices reported and ranked by foodservice operators* *(n=211) participating in an Australian national survey of healthy food retail support needs. Results include the proportion selecting each determinant, the proportion ranking it among the top three barriers/enablers, and mean proportion differences between comparison groups, along with 95% confidence intervals (CI).*

1. Percentage of foodservice operators selecting at least one enabler (n=211) or barrier (n=211)
2. Difference in mean proportions between implementers with experience and no experience with healthy food retail practices. Denominators include only those who selected at least one enabler (experienced n=157; no experience n=63) or barrier (experienced n=149; no experience n=62).
3. Difference in mean proportions between implementers with experience and no experience with healthy food retail practices. Denominators include only those who selected at least one enabler (experienced n=157; no experience n=63) or barrier (experienced n=149; no experience n=62).
