# Supplementary material for: Foodservice operator and implementation support practitioner needs for sustaining healthy food retail practices in Australian foodservice settings: findings from a national cross-sectional survey
Source: Int J Behav Nutr Phys Act. 2026 Aug 5;23:106. doi: 10.1186/s12966-026-01944-7 (PMC13729286; doi:10.1186/s12966-026-01944-7)
Supplement: Supplementary file 3 [file 12966_2026_1944_MOESM3_ESM.docx]

**Additional File 3:** Difference in preferred support options for sustaining healthy food retail practices ranked by foodservice operators with and without healthy food retail experience

| SUPPORT OPTION | % ranking determinant in Top 3  (95% CI) ^[1]^ | Difference in % ranking determinant in Top 3  (mean % difference, 95% CI) |
| --- | --- | --- |
|  |  | No experience (n=52) v. experienced (n=146) ^[2]^ |
| Making money by selling healthy options | 21 (16, 27) | -5 (-16, 6) |
| Help identifying and training a staff member to lead healthy changes in my outlet | 17 (12, 23) | 0 (-10, 10) |
| Support to plan healthy changes in a way that works for my outlet | 16 (12, 22) | +7 (-3, 17) |
| Access to external funding or financial incentives to support healthy changes | 15 (11, 21) | -3 (-13, 7) |
| Improving the skills of the staff to prepare healthier options | 15 (11, 21) | +6 (-4, 16) |
| Recipe modifications to deal with food allergies and intolerance while meeting nutrition standards | 15 (10, 20) | -4 (-14, 5) |
| Engaging customers in healthy options | 15 (10, 20) | -2 (-12, 7) |
| Support with getting feedback from customers | 14 (10, 19) | -5 (-15, 4) |
| Advice or support on labelling (e.g., traffic light labelling/health stars/ 'everyday' or 'occasional' labelling, high or low in a nutrient) or naming of menu items | 14 (10, 19) | -8 (-17, 2) |
| Support identifying new or alternate healthy products | 13 (9, 19) | -5 (-14, 4) |
| Advice on food or financial waste management | 12 (8, 17) | -1 (-10, 7) |
| Resources to promote the healthy food options to customers | 12 (8, 17) | -7 (-16, 1) |
| Support identifying new suppliers | 11 (7, 16) | **-9 (-17, 0)*** |
| Feedback on how my food outlet is performing against nutrition/food policy targets | 11 (7, 16) | +2 (-7, 10) |
| Support to connect with other retailers who have successfully made healthy changes | 11 (7, 16) | +4 (-4, 13) |
| Advice on setting the everyday price of items | 11 (7, 16) | +3 (-6, 11) |
| Advice on price promotions or special deals | 10 (7, 15) | +3 (-5, 11) |
| Help auditing the healthiness of our food environment, product offerings, or compliance with policy | 10 (6, 15) | -3 (-11, 5) |
| New equipment to prepare, store or display healthy foods and/or drinks | 8 (5, 13) | -1 (-8, 6) |
| Advice on planograms or stock display | 6 (3, 10) | **-7 (-14, -1)*** |
| Advice on marketing or advertising for the food outlet to customers | 6 (3, 10) | 0 (-6, 6) |
| * Statistically significant difference at p < 0.05 | | |

*This table presents support options that retailers reported wanting to access and perceived as helpful for sustaining healthy food retail practices in Australian foodservice settings. Results show the proportion of retailers ranking each support option among their top three supports for sustainment and mean proportion differences between comparison groups, with 95% confidence intervals (CI).*

1. Percentage of implementers ranking the support option among their top three based on importance for sustainment (n=198)
2. Difference in mean proportions between implementers with experience (n=146) and no experience with healthy food retail practices (n=52). Denominators include only those who selected at least one support option.
